# Visual and Linguistic Stimuli in the Remote Associates Test: A Cross-Cultural Investigation

**DOI:** 10.3389/fpsyg.2019.00926

**Published:** 2019-04-26

**Authors:** Teemu Toivainen, Ana-Maria Olteteanu, Vlada Repeykova, Maxim Likhanov, Yulia Kovas

**Affiliations:** ^1^Department of Psychology, Goldsmiths, University of London, London, United Kingdom; ^2^The International Centre for Research in Human Development, Tomsk State University, Tomsk, Russia; ^3^Tomsk State University, Tomsk, Russia; ^4^Sirius Educational Centre, Sochi, Russia

**Keywords:** Remote Associates Test, creativity, measurement, cross-cultural, visual stimuli

## Abstract

The Remote Associates Test (RAT) is a measure of associative ability, which is often regarded as essential for creative thinking. The most commonly used version of the test is the compound RAT. However, many RAT items do not translate directly in different languages. Additionally, a linguistic measure cannot be used to measure visual associative ability. A visual measure for associative ability that is similar to the RAT would be a useful tool for cross-cultural investigations of creativity. The present study investigated the relationship between the linguistic and a newly developed visual version of RAT in Russian and Finnish native speakers (for both samples *n* = 67). Both linguistic and visual measures showed good internal reliabilities in both samples (Cronbach’s α = 0.73–0.84). The mean score in the visual task was slightly higher for the Finnish sample. The correlation between the two measures was stronger in the Russian sample (*r* = 0.56) compared to the Finnish sample (*r* = 0.28). These results are discussed in relation to linguistic and cultural differences between the samples.

## Introduction

The Remote Associates Test (RAT) is a widely used measure in creativity research. The RAT was developed by Mednick (1962) to empirically test his associative theory of creativity. According to the theory, creative individuals are better at making remote associations in comparison to non-creative (Mednick, 1962). The originally proposed version of the RAT is to find a solution word for three stimuli words. According to Mednick (1962), the solution word can be associated with the stimuli by semantic association (e.g., chicken and egg), synonymy (e.g., chicken and coward) or formation of a compound word (e.g., spring chicken). The most commonly used version of the RAT is the compound Remote Associates Test (cRAT; Bowden and Jung-Beeman, 2003). In the cRAT the stimuli words form a compound word with the solution word. For example, for stimuli words “*cake*,” “*swiss*,” and “*cottage*,” a potential answer is “*cheese*,” because it creates compound words that have new meanings: “*cheesecake*,” “*swiss cheese*,” and “*cottage cheese*.” Traditionally, the cRAT has appealed to researchers as each item is held to have only one correct response, making scoring easy as well as taking limited space and time to administer (Bowden and Jung-Beeman, 2003; Lee et al., 2014). However, new computational approaches have shown that many cRAT stimuli words have more than one correct answer (Olteteanu and Falomir, 2015).

The cRAT has been used in several languages and has provided normative data for example in English (Bowden and Jung-Beeman, 2003), Dutch (Chermahini et al., 2012), and Japanese (Terai et al., 2013). Due to the language specific rules on forming compound words, the translation of the test items is often difficult if not impossible. Also, due to high demands of vocabulary in the cRAT, native speakers have been shown to have an advantage compared to second language speakers (Estrada et al., 1994). Additionally, some researchers have argued that the cRAT is limited as a measure of remote associational ability due to its overreliance on linguistic rules (Worthen and Clark, 1971).

Another variation of linguistic RAT is the functional RAT (fRAT; Worthen and Clark, 1971). As in the cRAT, participants are asked to come up with words that are associated with the three stimuli words. However, instead of creating compound words, the response word is connected to the stimuli with semantic associations. For example, for stimuli “*bait*,” “*pond*,” and “*tuna*,” the answer word can be “*fish*” (bait is used to catch fish, fish live in ponds and tuna is a type of fish). In the fRAT, it is likely that there are also other potential words that may connect the stimuli words semantically. A set of functional items has been created computationally (Olteteanu et al., 2018). Additionally, a recent extension of the fRAT is the visual Remote Associates Test (vRAT). In the vRAT, participants are asked to identify a concept that is semantically linked with three presented images (Olteteanu et al., 2015).

The vRAT has many advantages. Firstly, the use of visual stimuli in the vRAT overcomes limitations of language specificity for linguistic measures. The use of the vRAT instead of linguistic versions of the test may reduce the advantage of native speakers over second-language participants. Secondly, the use of vRAT in combination with linguistic RAT measures, can address questions relating to domain-specificity in creativity research. Mednick (1962) argued that his measure is domain-general but other researchers have proposed that the cRAT in particular is a domain-specific measure that taps into verbal abilities linked to a general intelligence factor (Kaufman et al., 2008).

The present study provides further information on the validity of different versions of the RAT as a measure of associative ability by investigating the relationship between visual and linguistic RAT measures in two samples of Russian and Finnish native speakers. A correlation of 0.37 between the cRAT and vRAT has been reported in an English speaking sample (*n* = 38; Olteteanu and Zunjani, 2019). The present study addressed the following questions:

(1) Is there a relation between the linguistic and visual RAT performance in Finnish?
(2) Is there a relation between the linguistic and visual RAT performance in Russian?
(3) Are these relations similar in the Russian and Finnish samples?

In addition, the study investigated potential difference in the vRAT between the Russian and Finnish samples. A mean difference in the visual task could be an indication of culture/language-specificity. For example, certain images could be more relevant in some cultures than in others.

## Methods

### Sample

The participants were members of general public, recruited via social media. Both Russian and Finnish samples had 67 participants (age range 18 to 69; see the Supplementary Material for details). The Russian sample included 17 males and 50 females, the Finnish sample 7 males and 60 females. *A priori* power analysis showed that a sample of 52 participants would be required to detect an effect of 0.37 (Olteteanu and Zunjani, 2019) with 80% power at significance level of 0.05.

### Measures

Same visual items were used for both samples (vRAT). The test included 46 items. For the development of visual items, see Olteteanu et al. (2015) for further details.

Translation of the English cRAT items (Bowden and Jung-Beeman, 2003) to Russian and Finnish was unsuccessful due to changes in the meanings of the words. Therefore, some Russian and all Finnish linguistic RAT (lingRAT) items were created for this study. Linguistic items and test forms (cRAT, fRAT) differed between the samples. In the Finnish sample, all 47 linguistic items were in the compound form (cRAT). In the Russian sample, 48 items were both in compound (cRAT) and functional (fRAT) forms. The use of different lingRAT stimulus sets was aimed to provide insights on the form of linguistic stimuli (compound vs. functional) in relation to the vRAT.

The study utilized 36 previously used Russian lingRAT items (Druzhinin, 1999). Twelve additional items, both compound and functional, were created by the research team. The items were tested by a group of native Russian speakers to make sure the items were commonly known (procedure similar to Chermahini et al., 2012). The lingRAT items were created in Finnish by the research team (procedure similar to Chermahini et al., 2012). However, no piloting was done prior to the present study. Examples of the measures (in English, Russian, and Finnish) are presented in Table 1. All Russian and Finnish lingRAT items are presented in the Supplementary Material.

**TABLE 1:** Example items of cRAT, fRAT, and vRAT.

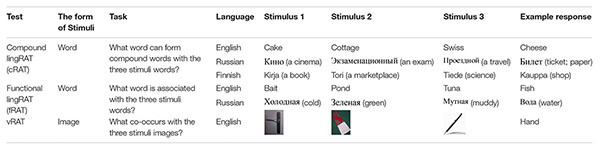

*lingRAT, linguistic RAT; vRAT, visual RAT; cRAT, compound RAT; fRAT, functional RAT.*

In all tasks, participants were asked to provide an answer word that is connected to stimuli. Also, participants were shown two practice items with example answers. No time limits for the tasks were set to replicate the procedure of the initial study (Olteteanu et al., 2015). In all tasks, participants could skip the items they did not have an answer for.

In addition to the responses (accuracy), reaction times (RT) were recorded for all items (see Supplementary Material). RTs longer than 400,000 ms (6 min and 40 s) were coded as outliers and imputed with the new series mean method in SPSS. The cut-off point was chosen to exclude extreme outliers at this pilot stage of the project. This will be redefined in the following studies, in which, with the additional data, we can make more informed decisions regarding the cut-off for the reaction times.

### Scoring

All responses (lingRAT and vRAT) were checked and scored by native Russian and Finnish speakers. This was to make sure that all correct answers were identified, since some of the items could have more than one correct answer. Correct answers were assigned 1 point, incorrect answers scored 0. The summed total was used as an Accuracy score for each participant.

## Results

Descriptive statistics and frequency distributions showed that all measures (RAT scores and RTs) were normally distributed. Table 2 presents descriptive statistics, internal reliabilities (Cronbach’s alpha), within sample correlations and the total mean time for the four measures (Russian vRAT, Russian lingRAT, Finnish vRAT, and Finnish lingRAT).

**TABLE 2:** Mean accuracy means (standard deviations); internal reliabilities (Alpha); skewness and kurtosis values; mean accuracy correlations; total mean times; and total mean time correlations for the vRAT and lingRAT in Russian and Finnish samples.

		M (SD)	Alpha	Skewness	Kurtosis	Accuracy correlation	M total time (in minutes)	Total time correlation
Russian	vRAT	24.6 (6.8)	0.79	−0.61	1.30	0.56**	14.40 (5.8)	0.47**
	lingRAT	26.6 (6.9)	0.83	−0.76	0.71		18.83 (8.3)	
Finnish	vRAT	29.2 (7.1)	0.84	−1.99	5.57	0.28*	14.07 (6.49)	0.46**
	lingRAT	21.6 (5.3)	0.73	0.37	0.45		29.25 (13.6)	

**p < 0.01 and *p < 0.05. *n* = 134; n_russian_ = 67, n_finnish_ = 67.

The correlation between the lingRAT and vRAT in the Russian sample was *r*(65) = 0.56, *p* < 0.001, and *n* = 67, and in the Finnish sample it was *r*(65) = 0.28, *p* = 0.02, and *n* = 67. The difference between sample-specific correlations was statistically significant (Fisher’s r-to-z transformation *z* = 1.95 and *p* = 0.03). Additionally, there was a significant mean difference in vRAT [*t*(132) = −3.78 and *p* < 0.001] between the Russian and Finnish samples. The total reaction times (sum of RTs for each item) were positively correlated between lingRAT and vRAT total scores for both Russian [*r*(65) = 0.47 and *p* < 0.001] and Finnish [*r*(65) = 0.46 and *p* < 0.001] samples. The difference in correlations was non-significant (Fisher’s r-to-z transformation *z* = −0.07).

## Discussion

The present study was the first to explore the relationship of linguistic and visual stimuli in the RAT in Russian and Finnish samples. Correlations between accuracy scores in the linguistic (cRAT + fRAT) and visual (vRAT) tasks differed between the samples: correlation was moderate in the Russian sample and weak in the Finnish sample. For the RT measure, a very similar moderate correlation was found in both samples.

The difference in the lingRAT stimuli sets may influence the accuracy correlation between the lingRAT and vRAT. Finnish items were all compound words whereas Russian items were a combination of both compound and functional items (30 functional items). In the vRAT, all items were the same for both groups. Since the vRAT is based on semantic associations (same as linguistic fRAT items), the higher correlation in the Russian sample may reflect that the similar strategy could be used to solve items in lingRAT and vRAT. Conversely, the lower correlation in the Finnish sample could be due to differences in measures. Whereas the vRAT tapped into semantic associations, performance in the Finnish lingRAT (all compound items) was more related to linguistic ability to form compound words than it was in the Russian sample.

Alternatively, the difference between the correlations may also indicate language-specific features of how compound words are created. Due to different linguistic rules in Russian and Finnish, it may be that language specific grammatical constraints direct the selection of the words that can be used to form compound words. For example, if in Russian fRAT items a stimulus word is an adjective, it will have the appropriate grammatical gender in congruence with the solution word. Potentially this will also constrain the search space for the correct solution word.

Overall, the two samples performed very similarly in the visual RAT. The frequency distributions were largely overlapping with similar ranges. However, there was a small mean difference in the vRAT between the samples. Future research is needed to explore whether this difference stems from methodological limitations or some culture/language specificity. The observed difference may reflect culture-specificity of certain items, when some concepts (images) may be more familiar in certain cultures. For example, a picture of Poseidon is recognizable only to participants with knowledge on Greek mythology.

Different proportions of linguistic test items was also a limitation in the study. In our future work we will address this by creating comparable stimulus sets to investigate the relationships of lingRAT (cRAT, fRAT) and vRAT within the samples. Additionally, we will also investigate the psychometric properties of the linguistic and visual items in more detail. Future studies should also employ the same stimuli, both in linguistic or visual form, to explore their role in associative processing. Eventually, future studies will help to further develop a valid vRAT measure that can be used in cross-cultural studies.

The findings of the present study show promise in the use of a vRAT across populations with different native languages. They also show that linguistic and cultural specificity may influence RAT performance. Using linguistic and visual remote association tests in cross-cultural context will lead to better understanding of the cognitive processes underlying creativity.

## Ethics Statement

This study was carried out in accordance with the recommendations of Goldsmiths’ (University of London) Ethics Committee’ with written informed consent from all subjects. All subjects gave written informed consent in accordance with the Declaration of Helsinki. The protocol was approved by the Goldsmiths’ (University of London) Ethics Committee.

## Author CONTRIBUTIONS

TT and AMO designed the study and wrote the manuscript. TT, AMO, VR, and ML created the test items. AMO and YK supervised all aspects of the study. All authors reviewed the manuscript.

## Conflict of Interest Statement

The authors declare that the research was conducted in the absence of any commercial or financial relationships that could be construed as a potential conflict of interest.
