# Supplementary material for: Visual and Linguistic Stimuli in the Remote Associates Test: A Cross-Cultural Investigation
Source: Front Psychol. 2019 Apr 26;10:926. doi: 10.3389/fpsyg.2019.00926 (PMC6498948; doi:10.3389/fpsyg.2019.00926)
Supplement: Supplementary file 1 [file Table_1.DOCX]

**Supplementary Material 1. Items, responses and reaction times for the Russian linguistic RAT.**

| Item | Stimuli | Response | Correct answers (n = 67) | Mean RT and sd in seconds for correct answers | Mean RT and sd in seconds for all answers |
| --- | --- | --- | --- | --- | --- |
| Training 1 | громкая, правда, медленно | Говорить |  |  |  |
| Training 2 | холодная, зелень, мутная | Вода |  |  |  |
|  |  |  |  |  |  |
|  | прошлое, море, друзья | Вспомнить | 19 | 25.50 (28.1) | 24.99 (30.5) |
|  | зоркий, ресница, стеклянный | Глаз | 60 | 13.71 (19.4) | 12.68 (18.6) |
|  | свежая, английская, новости | Газета | 51 | 11.80 (18.0) | 13.42 (18.3) |
|  | кино, экзамен, проездной | Билет | 52 | 12.72 (13.4) | 16.85 (19.3) |
|  | комната, положение, река | Войти | 4 | 34.82 (25.8) | 52.44 (69.0) |
|  | трудное, истекло, золото | Время | 57 | 10.59 (7.9) | 11.99 (95.6) |
|  | мундир, городок, билет | Военный | 33 | 27.90 (42.4) | 28.50 (35.5) |
|  | неожиданно, человек, улица | Встреча | 45 | 18.78 (21.8) | 21.10 (22.3) |
|  | холодная, дым, жестокая | Война | 19 | 30.20 (54.6) | 34.17 (39.4) |
|  | умная, косы, свежая | Голова | 25 | 33.33 (44.5) | 32.44 (39.8) |
|  | прошлый, время, трудный | Год | 61 | 18.45 (17.9) | 18.33 (17.9) |
|  | дедушка, очки, добрая | Бабушка | 40 | 20.23 (24.5) | 23.61 (29.7) |
|  | долго, вечер, друзья | Ждать | 36 | 24.74 (29.1) | 24.92 (32.3) |
|  | плохо, глаза, море | Видеть | 16 | 21.03 (13.6) | 25.69 (31.3) |
|  | слон, дом, великан | Большой | 40 | 23.14 (61.3) | 22.73 (48.9) |
|  | навсегда, домой, назад | вернуться | 37 | 22.48 (27.1) | 19.22 (21.3) |
|  | случайная, горы, долгожданная | Встреча | 36 | 16.57 (16.9) | 22.77 (28.9) |
|  | вечерняя, бумага, стенная | Газета | 47 | 18.09 (30.2) | 22.08 (30.8) |
|  | обратно, родина, путь | Вернуться | 26 | 12.61 (7.1) | 15.02 (13.5) |
|  | далеко, слепой, будущее | Смотреть | 23 | 19.80 (22.0) | 30.21 (35.2) |
|  | народная, страх, мировая | Война | 61 | 12.95 (15.6) | 14.41 (17.9) |
|  | деньги, билет, свободное | Время | 20 | 25.58 (24.0) | 18.57 (18.5) |
|  | человек, погоны, завод | Военный | 25 | 21.20 (15.0) | 25.56 (22.3) |
|  | дверь, доверие, быстро | Войти | 6 | 13.36 (6.0) | 35.16 (33.1) |
|  | друг, город, круг | Родной | 25 | 22.41 (14.2) | 37.05 (37.9) |
|  | поезд, купить, бумажный | Билет | 63 | 9.49 (5.4) | 9.41 (55.2) |
|  | цвет, заяц, сахар | Белый | 52 | 13.96 (18.2) | 14.84 (18.0) |
|  | ласковая, морщины, сказка | Бабушка | 58 | 12.04 (9.3) | 16.80 (33.7) |
|  | детство, случай, хорошее | Настроение | 29 | 22.09 (45.3) | 35.88 (60.7) |
|  | воздух, быстрая, свежая | Струя | 24 | 26.26 (24.0) | 29.05 (32.5) |
|  | певец, Америка, тонкий | Голос | 34 | 30.46 (35.1) | 42.42 (51.3) |
|  | тяжелый, рожденье, урожайный | Год | 59 | 15.10 (16.5) | 15.92 (17.1) |
|  | много, чепуха, прямо | Говорить | 37 | 14.05 (10.3) | 16.75 (15.3) |
|  | кривой, очки, острый | глаз | 38 | 17.86 (17.5) | 21.97 (36.5) |
|  | садовая, мозг, пустая | Голова | 34 | 15.04 (13.9) | 29.53 (34.1) |
|  | гость, случайно, вокзал | встреча | 37 | 22.75 (26.5) | 27.64 (33.7) |
|  | Броня, пуля, дыра | Бронебойный | 27 | 18.79 (14.4) | 21.54 (20.6) |
|  | Вода, течь, высота | Водопад | 50 | 15.69 (21.9) | 22.52 (39.8) |
|  | Вода, дыра, кружить | Водоворот | 48 | 24.05 (37.5) | 22.96 (32.4) |
|  | Птица, крутить, шея | Вертишейка | 1 | 8.76 (0.0) | 24.15 (25.4) |
|  | Птица, нести, весть | Буревестник | 31 | 21.64 (34.4) | 24.32 (35.1) |
|  | Собака, охота, волк | Волкодав | 19 | 29.21 (33.7) | 27.90 (26.4) |
|  | Летать, винт, пассажир | Вертолет | 64 | 10.25 (7.3) | 10.56 (77.5) |
|  | Мерить, насекомое, вода | Водомерка | 38 | 15.83 (20.9) | 32.38 (49.4) |
|  | Дождь, календарный, растущий | Месяц | 24 | 28.82 (24.0) | 32.47 (30.7) |
|  | День, заяц, цвет | Серый | 44 | 15.25 (21.8) | 16.17 (18.7) |
|  | Невеста, жених, кольцо | Свадьба | 62 | 9.43 (8.5) | 11.48 (18.4) |
|  | Подсолнух, солнце, масло | Цветок | 43 | 17.38 (23.0) | 18.08 (20.3) |
