# Supplementary material for: Visual and Linguistic Stimuli in the Remote Associates Test: A Cross-Cultural Investigation
Source: Front Psychol. 2019 Apr 26;10:926. doi: 10.3389/fpsyg.2019.00926 (PMC6498948; doi:10.3389/fpsyg.2019.00926)
Supplement: Supplementary file 2 [file Table_2.DOCX]

**Supplementary Material 2. Items, responses and reaction times for the Finnish linguistic RAT.**

| Item | Stimuli | Response | Correct answers (n = 67) | Mean RT in seconds and sd for correct answers | Mean RT in seconds and sd for all answers |
| --- | --- | --- | --- | --- | --- |
| Training 1 | Kauppa, hylly, paino | Kirja |  |  |  |
| Training 2 | Nyrkki, tie, vohveli | rauta |  |  |  |
|  |  |  |  |  |  |
|  | Alue, haju, sänky | vesi | 21 | 32.45 (32.49) | 46.37 (45.4) |
|  | Apulainen, vaali, käännös | toimisto | 14 | 50.66 (58.96) | 41.62 (40.9) |
|  | Asfaltti, koti, pako | tie | 41 | 13.85 (11.04) | 22.38 (26.6) |
|  | Asia, lyhty, ruutu | paperi | 12 | 54.79 (27.60) | 61.71 (70.2) |
|  | Etä, kauppa, paikka | kauppa | 51 | 11.08 (6.59) | 17.68 (18.9) |
|  | hanhi, hätä, hirviö | meri | 17 | 41.89 (42.86) | 57.05 (65.5) |
|  | Harja, vuode, suoja | vaate | 18 | 30.08 (27.11) | 33.86 (31.2) |
|  | henkilö, paikka, vero | auto | 37 | 19.55 (11.92) | 24.86 (21.3) |
|  | Ikkuna, tiili, väli | katto | 13 | 40.11 (43.97) | 29.54 (31.5) |
|  | Juhla, voitto, kaari | riemu | 19 | 41.53 (48.00) | 55.32 (63.2) |
|  | Kala, avio, arpa | onni | 45 | 14.73 (11.24) | 20.30 (17.6) |
|  | Kasa, koulu, esine | puu | 6 | 86.4 (85.5) | 68.44 (65.9) |
|  | Käsittely, vero, sosiaali | virasto | 16 | 21.96 (13.84) | 26.86 (19.3) |
|  | Kastike, joki, soija | kauha | 3 | 54.49 (34.27) | 54.99 (50.6) |
|  | Kauha, hiutale, riisi | puuro | 59 | 14.62 (14.57) | 17.15 (16.9) |
|  | Kaula, sukellus, heiluri | Kello | 35 | 19.92 (17.38) | 28.21 (28.1) |
|  | Kauppa, tie, kalastaja | Kylä | 15 | 40.37 (47.77) | 45.48 (50.7) |
|  | Kerros, vesi, pinna | sänky | 61 | 13.37 (8.48) | 14.05 (8.4) |
|  | Kone, kello, aika | peli | 18 | 62.57 (70.80) | 54.51 (55.1) |
|  | Kukka, tori, tiede | kauppa | 41 | 33.62 (38.96) | 33.62 (38.7) |
|  | Kuoro, viini, alku | kirkko | 11 | 41.11 (40.4) | 49.35 (53.7) |
|  | Laukku, nahka, turva | vyö | 23 | 50.03 (74.39) | 58.98 (83.1) |
|  | Lehti, vene, aitta | puu | 42 | 26.28 (28.1) | 36.65 (44.5) |
|  | Leike, väli, paino | lehti | 21 | 34.77 (28.9) | 33.22 (32.4) |
|  | Leikki, puhelin, ryhmä | seksi | 9 | 57.23 (60.4) | 41.39 (41.0) |
|  | Lukko, aisti, herne | haju | 45 | 25.13 (23.82) | 32.88 (27.2) |
|  | Metsä, vara, kunto | Mies | 11 | 62.02 (103.12) | 49.96 (56.9) |
|  | Mieli, kunta, tuomio | valta | 10 | 50.45 (39.25) | 64.70 (62.4) |
|  | Mini, kahvi, jakkara | baari | 13 | 42.85 (47.29) | 57.94 (54.0) |
|  | Muisti, synti, viini | Lista | 9 | 52.18 (55.61) | 61.23 (51.7) |
|  | Pää, lista, öljy | ruoka | 12 | 58.30 (63.07) | 50.16 (49.3) |
|  | Paisti, aamu, sokeri | pala | 53 | 19.94 (21.48) | 25.96 (27.2) |
|  | Pelto, sämpylä, lese | Vehnä | 61 | 17.07 (15.53) | 17.34 (15.0) |
|  | Poika, adoptio, ihme | lapsi | 54 | 13.67 (12.09) | 17.67 (24.9) |
|  | Ravintola, kaula, veto | ketju | 26 | 37.55 (77.62) | 52.71 (72.9) |
|  | Rotu, kesä, kolli | kissa | 66 | 12.00 (15.08) | 12.11 (14.9) |
|  | Rotu, kesä, kolli | pöytä | 46 | 21.02 (25.98) | 20.83 (23.1) |
|  | Sali, kevät, suku | juhla | 63 | 14.83 (15.36) | 15.00 (15.2) |
|  | Sarja, kulta, veto | ketju | 18 | 25.78 (21.91) | 38.93 (34.5) |
|  | Sarja, palvelu, hätä | numero | 40 | 14.42 (9.41) | 21.31 (18.5) |
|  | Tentti, velka, kirja | kirja | 45 | 23.47 (39.21) | 26.91 (38.1) |
|  | Tölkki, hana, makkara | olut | 55 | 20.16 (22.98) | 26.13 (32.0) |
|  | Vanki, salama, sota | sota | 10 | 55.91 (36.7) | 68.46 (64.2) |
|  | Verho, lippu, juoppo | tanko | 51 | 16.43 (16.51) | 25.73 (32.6) |
|  | Vesi, kunto, apu | Pyörä | 18 | 49.82 (65.75) | 33.32 (39.0) |
|  | Vyö, kaali, joulu | ruusu | 40 | 21.86 (30.80) | 33.15 (39.5) |
|  | Yhdys, vuoro, tunnus | sana | 55 | 10.81 (84.5) | 13.07 (10.5) |
