# Supplementary material for: Visual and Linguistic Stimuli in the Remote Associates Test: A Cross-Cultural Investigation
Source: Front Psychol. 2019 Apr 26;10:926. doi: 10.3389/fpsyg.2019.00926 (PMC6498948; doi:10.3389/fpsyg.2019.00926)
Supplement: Supplementary file 3 [file Table_3.docx]

**Supplementary Material 3. Frequencies of Russian and Finnish participants in different age categories.**

| Age range | Russian sample | Finnish sample |
| --- | --- | --- |
| 18-19 | 3 | 0 |
| 20-29 | 47 | 15 |
| 30-39 | 15 | 16 |
| 40-49 | 1 | 19 |
| 50-59 | 1 | 14 |
| 60-69 | 0 | 3 |
